# Non-Responder to Inclisiran and Evolocumab—A Female Patient with Heterozygous Familial Hypercholesterolemia and Statin Intolerance

**DOI:** 10.3390/diseases14040125

**Published:** 2026-04-01

**Authors:** Paweł Muszyński, Małgorzata Chlabicz, Joanna Kruszyńska, Katarzyna Wilk-Śledziewska, Piotr Kazberuk, Dominika Musiałowska, Monika Groth, Kinga Dudzińska

**Affiliations:** 1 Department of Cardiology, Lipidology and Internal Diseases, Medical University of Bialystok, Żurawia 14, 15-569 Bialystok, Poland; 2 Department of Invasive Cardiology, Medical University of Bialystok, M. Skłodowskiej-Curie 24A, 15-276 Bialystok, Poland

**Keywords:** lipid disorders, familiar hypercholesterolemia, pharmacotherapy, lipoprotein(a)

## Abstract

Despite the availability of numerous lipid-lowering agents, the treatment of lipid disorders remains a public health challenge. A substantial portion of patients, especially those with severe dyslipidemia or familial hypercholesterolemia (FH), fail to achieve the LDL-C goal. The leading causes of suboptimal LDL-C control include underprescription and poor adherence; however, in rare cases, it may result from an unusual biological response to treatment. In the presented case, a 78-year-old female with a history of transient ischemic attack and myocardial infarction was diagnosed with a heterozygous variant of FH and true statin intolerance following trials of simvastatin, rosuvastatin and pitavastatin. Initially, inclisiran was added to ezetimibe, leading to an unexpected increase in LDL-C. Due to the patient’s refusal of another statin re-challenge and the unavailability of bempedoic acid, nutraceuticals were introduced. After 6 months, inclisiran was discontinued because only a 22% reduction in LDL-C was achieved, likely attributable to the nutraceutical’s effect. Another PCSK9 inhibitor, evolocumab, was subsequently initiated. Shortly after the treatment onset, the patient complained of paraesthesia in the upper extremities and discontinued therapy. LDL-C levels increased by 7% after one month of treatment with evolocumab. The patient refused treatment with lipid apheresis. Possible causes of poor response to PCSK9 inhibitors include elevated lipoprotein(a) and FH.

## 1. Introduction

Atherosclerotic cardiovascular disease remains the leading cause of mortality worldwide, despite the advances in prevention and improved availability of diagnostic tools [1]. Among the core modifiable risk factors are lipid disorders. Current management focuses on widespread life-style modifications and lipid-lowering therapies. Treatment should be initiated with a high-potency statin, and if the low-density lipoprotein (LDL) cholesterol goal is not achieved, ezetimibe and proprotein convertase subtilisin/kexin type 9 (PCSK9) inhibitors with proven benefit should be added [2,3]. PCSK9 is a protein that promotes the degradation of LDL receptors (LDL-R), which binds with LDL cholesterol (LDL-C) by its particle apolipoprotein B (APOB), thereby reducing LDL cholesterol (LDL-C) clearance. Two therapeutic classes of PCSK9 inhibitors (PCSK9i) are currently available: monoclonal antibodies (evolocumab, alirocumab), which bind to PCSK9, and small interfering RNA (siRNA) therapy (inclisiran), which suppresses PCSK9 synthesis [1,2,3] (Table 1).

Despite the availability of effective medications, the majority of patients fail to reach the recommended LDL-C goals. The principal causes include the underprescription of medications and low adherence/discontinuation of the treatment.

Over the past two decades, prescriptions for statins and other lipid-lowering therapies have increased. Among patients with severe dyslipidemia, approximately 60–70% have been prescribed statins. Despite this, overall statin use remains relatively low, likely due to patient-related factors. Even in individuals at high risk of atherosclerotic cardiovascular disease (ASCVD), only about half of patients continue statin therapy after one year, and this proportion drops to around 20% after five years. Among those who do not adhere to treatment, more than half report concerns about potential side effects or prefer alternative approaches, such as dietary changes and exercise, to lower cholesterol level. Additionally, negative media coverage has been linked to reduced adherence. Together, these patient-related factors contribute to lower statin use among those who are eligible for treatment. It has been reported that, even among patients with severe dyslipidemia (LDL-C over 190 mg/dL), only 34% of patients were taking statins [6].

Additionally, severe dyslipidemia has been associated with a higher prevalence of other cardiovascular risk factors such as hypertension, obesity, and diabetes, which act together to increase the risk of developing ASCVD tremendously. Furthermore, severe dyslipidemia may be considered an indicator of underlying familial hypercholesterolemia. Familial hypercholesterolemia is 23 times more common in individuals with severe dyslipidemia than in the general population. FH is a common genetic disease, with a prevalence of 1/311 worldwide, causing a significant burden for public health by increasing the occurrence of premature atherosclerotic cardiovascular diseases. The FH is one of most prevalent comorbidities among patients with already-established ASCVD (1/17 patients) [7,8]. Classical causes include monogenic autosomal dominant mutations in LDL-R, APOB and PCSK-9 (Table 2). The majority of cases occur as a heterozygotic mutations with a prevalence ranging from 1/67 to 1/500. Homozygous FH mutations are extremely rare, occurring in 1/1000 000 [9]. Other uncommon mutations include LDL receptor adaptor protein 1 (LDLRAP1), apolipoprotein E (APOE) and diseases with an FH phenotype such as cerebrotendinous xanthomatosis, cholesterol ester storage disease, and sitosterolemia [9].

Currently, the majority of patients are diagnosed basing on the Dutch Lipid Clinic Network Score (DLCNS). However, the genetic mutations can currently be found only in 40–60% of patients with clinical diagnosis of familial hypercholesterolemia [9,10]. The genetic assessment is crucial for cascade screening among relatives of the probands, which can additionally explain the unusual response and allow for a personalized approach [11,12].

Despite increasing awareness of familial hypercholesterolemia, patients with FH are often underdiagnosed, but even when identified, they are frequently undertreated [1]. The problem of low adherence to statin therapy—often driven by patient-reported intolerance and discontinuation—is even more pronounced among patients with familial hypercholesterolemia. In the Familial Hypercholesterolemia Studies Collaboration registry including 61 612 patients, only 3% achieved the LDL-C target of 70 mg/dL. Worldwide, patients with familial hypercholesterolaemia are predominantly treated with statin monotherapy, although only a small proportion (approximately 14%) receive high-intensity doses such as atorvastatin or rosuvastatin. The use of combination therapy—adding ezetimibe to statins—or triple therapy including PCSK9 inhibitors significantly improves the likelihood of achieving LDL cholesterol targets [13].

The possible causes of failure to achieve the LDL-C target include higher baseline LDL-C or variable biological response to lipid-lowering therapies. However, real-world data suggest that the prescription of PCSK9i can increase the proportion of patients reaching LDL-C goal up to 43.3% for heterozygous FH and 37.5% among those with homozygous FH [14]. However, a small subset of individuals demonstrates an unusual or absent response to these therapies (true unusual response in 7.5% of patients) [15].

## 2. Case Presentation

A 78-year-old female with hypertension, chronic kidney disease and a history of transient ischemic attack (three years prior) and myocardial infarction (MI) treated with percutaneous coronary intervention of the right coronary artery and left anterior descending artery at the age of 78 years, with carotid artery atherosclerosis and significant stenosis of left renal and left subclavian arteries was referred to a highly specialized Cardio-Lipid Center for optimization of pharmacological therapy due to statin intolerance.

Her medical history was notable for intolerance to simvastatin, rosuvastatin (administered as a high-potency statin during MI with intolerance during hospitalization), and pitavastatin at the lowest available dose prescribed upon discharge after MI. Physical examination was unremarkable apart from the patient being overweight (BMI 28 kg/m^2^). The initial low-density lipoprotein cholesterol (LDL-C) concentration was 265 mg/dL (6.9 mmol/L), corresponding to 5 points on the Dutch Lipid Clinic Network Score (DLCNS). Treatment with pitavastatin 2 mg combined with ezetimibe 10 mg reduced LDL-C to 203 mg/dL (5.2 mmol/L; 23% reduction after one month). However, the patient reported intolerance, and pitavastatin was discontinued.

Evaluation with the Statin-Associated Muscle Symptom Clinical Index (SAMS-CI) indicated a high probability of true statin intolerance (11 points). The LDL-C during ezetimibe monotherapy remained elevated at 219 mg/dL (5.7 mmol/L), and lipoprotein(a) (Lp(a)) concentration was 73.8 mg/dL (159 nmol/L).

Due to the confirmed statin intolerance after re-challenge, therapy with small interfering RNA targeting proprotein convertase subtilisin/kexin type 9 (PCSK9)—inclisiran—was initiated. Due to the DLCNS (5 points) and suspicion of familial hypercholesterolemia (FH), genetic testing and family screening were recommended for individuals with LDL-C > 190 mg/dL. After three months of inclisiran treatment, LDL-C paradoxically increased to 250 mg/dL (6.5 mmol/L).

Because of the patient’s very high cardiovascular risk, another statin re-challenge was proposed (due to the unavailability of bempedoic acid). The patient declined but agreed to treatment with nutraceuticals (a combination of monacolin K and *Citrus bergamia* extract). The family screening revealed elevated LDL-C (>95th percentile) in the patient’s son (220 mg/dL; 5.7 mmol/L). Genetic testing identified a heterozygous APOB mutation [NM_0003843.3(APOB):c.10580G>A(p.Arg3527Gln)Chr2(hg38):21006288C>T]. Re-evaluation using the DLCNS yielded 14 points, confirming the diagnosis of FH.

After six months of combined therapy with inclisiran, ezetimibe, and nutraceuticals, the patient’s LDL-C decreased to 193 mg/dL (12% reduction since inclisiran initiation) and further to 159 mg/dL (27% reduction) after seven months. However, treatment was discontinued per local regulations, which require at least a 30% LDL-C reduction. Therapy was then switched to the PCSK9 inhibitor evolocumab, but LDL-C increased by 7% to 170 mg/dL after one month. 

**Figure 1:**
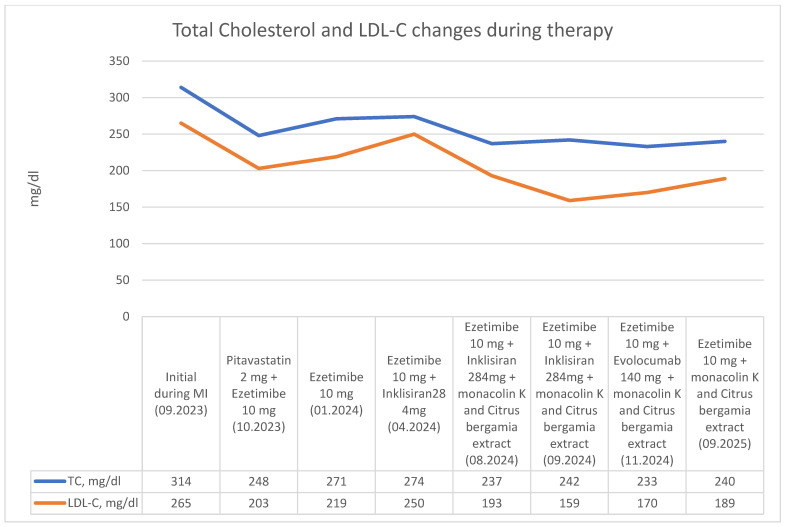
Total cholesterol and LDL-C changes during therapy.

The patient declined further therapeutic options, including LDL-C apheresis. Shortly after starting evolocumab, she reported paresthesia of the upper extremities. Neurological evaluation, including head and spine CT, attributed these symptoms to spondylosis. Nevertheless, the patient discontinued treatment after 2 months, which led to improvement of symptoms within several weeks. The summary of Total cholesterol and LDL-C changes during therapy is showed in Figure 1. 

## 3. Discussion

Our case report illustrates the complexity of managing a patient with familial hypercholesterolemia (FH), multidrug intolerance, and insufficient response to PCSK9 inhibitors (PCSK9i). Statin intolerance and the lack of efficacy of both inclisiran and evolocumab resulted in poor LDL-C control in a patient after MI with extremely high cardiovascular risk (LDL-C goal < 40 mg/dL) [3].

Statins have consistently demonstrated a favorable safety profile in multiple high-quality studies. Despite this, statin use among patients continues to be suboptimal. Reduced adherence to statin therapy has been observed in both primary and secondary prevention of cardiovascular disease. Muscle-related symptoms are frequently reported as a reason for discontinuing treatment. However, evidence from randomized trials indicates that only a slight increase in muscle symptoms is attributable to statins, with the majority of reported symptoms (>90%) unlikely to be caused by the medication. Overall, statin intolerance is reported in approximately 5–10% of patients initiating treatment. Nevertheless, in rare cases, statins may lead to serious muscle-related adverse effects, such as myopathy or rhabdomyolysis. The likelihood of adverse events appears to rise with more intensive statin therapy [16].

Establishing the diagnosis of statin intolerance should follow a thorough evaluation, including measurement of creatine kinase and alanine aminotransferase levels, as well as structured de-challenge and re-challenge attempts. Statins remain among the most effective lipid-lowering drugs, offering substantial pleiotropic benefits and a favorable cost-effectiveness profile [16]. In our patient, three different statins—simvastatin, rosuvastatin, and pitavastatin—were tried at the lowest available doses, fulfilling the criteria for true statin intolerance, which was further confirmed by a high SAMS-CI score (11 points). Although the European Society of Cardiology (ESC) guidelines recommend bempedoic acid for statin-intolerant patients to achieve LDL-C targets, it could not be initiated in our patient due to limited availability in Poland, primarily related to reimbursement restrictions and reduced accessibility. This gap between guideline recommendations and real-world practice may contribute to suboptimal LDL-C goal attainment, particularly in high-risk populations [3].

One of the possible explanations for the suboptimal response to therapy is homozygous FH; however, this was excluded in the present case [17]. Non-responders are frequently observed among patients with statin intolerance [18]. In a registry by Mulder et al., inclisiran achieved greater LDL-C reduction when used in combination with a statin (45% vs. 38%) [18]. Additionally, they reported withdrawal from therapy due to insufficient response in 7.7% of patients [18]. The true unusual response to PCSK9i occurs in approximately 7.5% of patients, and risk factors of such reaction include FH and the absence of concomitant ezetimibe use [15].

The first reported case of non-responsiveness to PCSK9 inhibition with both monoclonal antibody and RNA-interference therapy was published in 2024 [19]. The possible causes of nonresponse suggested by the authors were elevated lipoprotein(a) (Lp(a)) levels and rare genetic disorders such as sitosterolemia; however, the results of genetic evaluation were not available at the time of publication [19]. In contrast, in our case, next-generation sequencing identified a heterozygous APOB variant associated with FH (p.Arg3527Gln). The same mutation has been reported in two other patients with poor response to PCSK9i, both successfully treated with lipoprotein apheresis [20]. However, our patient has thus far declined lipoprotein apheresis.

Elevated Lp(a) concentrations have also been shown to attenuate LDL-C reduction achieved with PCSK9i. In a pooled analysis of evolocumab trials, patients with Lp(a) > 125 nmol/L experienced smaller LDL-C reductions (−56.2% vs. −61.7%) [21]. That can be caused by the inaccuracy of LDL-C measurement by homogeneous direct assays and gel-permeation HPLC (direct assessment) in individuals with elevated Lp(a) [4]. Thus, the effect on the LDL-C-lowering properties of medications not influencing the Lp(a) concentration can be alternated.

We suggest that all patients demonstrating inadequate or absent response to PCSK9i therapy undergo comprehensive evaluation, including confirmation of treatment adherence, assessment of possible drug interactions with lipid-lowering agents, measurement of Lp(a), and genetic testing.

In the present case, the modest LDL-C reduction may have resulted from adjunctive therapy with nutraceuticals containing monacolin K and *Citrus bergamia* extract, which are supported by local lipid-management guidelines [22]. However, both the current cardiovascular prevention and dyslipidemia guidelines by the ESC and European Atherosclerosis Society emphasize that nutraceuticals without proven clinical benefit and safety should not be routinely prescribed [2,3,23]. Additionally, the recent American College of Cardiology/American Heart Association Guideline on the Management of Dyslipidemia also does not recommend using supplements due to limited data regarding the benefits [24]. In our patient, they were considered a last-resort option after refusal of another statin re-challenge and following evidence of an unusual response to inclisiran.

## 4. Conclusions

This case highlights the complexity of managing familial hypercholesterolaemia in the setting of statin intolerance and variable response to advanced lipid-lowering therapies. Despite the availability of multiple treatment options, achieving LDL-C targets remains challenging due to biological variability, genetic factors, and limited access to novel therapies. Genetic variants, including APOB mutations, as well as elevated lipoprotein(a) concentrations, may contribute to a reduced LDL-C-lowering response to inclisiran or PCSK9 inhibitors. The recognition of potential non-responders to PCSK9-targeted treatments and the incorporation of genetic testing may facilitate a more individualized therapeutic approach. Further research is warranted to better understand the mechanisms of treatment resistance and improve outcomes in this high-risk population.

## Figures and Tables

**Table 1:** Summary of lipid-lowering medication influencing LDL-C [4,5].

Class	Effect	Mechanism
Statins, e.g., Atorvastatin, Rosuvastatin, Pitavastatin	Decreased synthesis of LDL-C	Competitively inhibits HMG-CoA reductase
Cholesterol absorption inhibitor, e.g., Ezetimibe	Decreased absorption of cholesterol	Decreases intestinal and biliary sterol absorption via blocking NPC1L1 transporter protein
ATP citrate lyase inhibitor e.g., Bempedoic acid	Decreased synthesis of LDL-C	Inhibits ATP citrate lyase leading to decreased cholesterol synthesis
PCSK9 inhibitor: monoclonal antibodies, e.g., Alirocumab, Evolocumab	Increased degradation of LDL-C	Binding the circulating PCSK-9 leading to limitation of LDL-R degradation
PCSK9 inhibitor: small interfering RNA, e.g., Inclisiran	Increased degradation of LDL-C	Causes degradation of mRNA for PCSK-9 leading to limitation of LDL-R degradation
ANGPTL3 inhibitor, e.g., Evinacumab	Decreased synthesis of LDL-C	Inhibits ANGPTL3 increasing remnant lipoprotein clearance, reducing conversion of VLDL remnants into LDL-C
Microsomal TG transfer protein inhibitor, e.g., Lomitapide	Decreased synthesis of LDL-C	Inhibits microsomal triglyceride transfer protein preventing creation of chylomicrons and VLDL leading to limitation of conversion to LDL-C

**Table 2:** Summary of FH-related mutations [9,10].

Gene	Percent FH Attributed to Gene	Inheritance	Type of Mutation
LDL-R	60–90%	Semidominant	Loss-of-function
APOB	~5%	Semidominant	Loss-of-function
PCSK-9	Up to 3%	Semidominant	Gain-of-function
LDLRAP1	Rare	Recessive	Double loss-of-function
APOE	Very rare	Autosomal dominant	-

## Data Availability

The original contributions presented in this study are included in the article. Further inquiries can be directed to the corresponding author.
